# Food Adulteration

**Published:** 1910-06-04

**Authors:** 


### FOOD ADULTERATION.

It is a relief to find in an official publication some indication that tradesmen realise not only the impropriety, but the unwisdom of dealing in faked and adulterated foodstuffs. This is all the more satisfactory in view of the great attention which the lay Press has recently paid to alarmist statements as to the extent to which the sale of such foods is carried on. The annual report of the Intelligence Division of the Board of Agriculture, which has just been issued, records several instances in which associations of traders have drawn the attention of the Board to articles of food suspected to be adulterated. It is a very healthy and promising sign when shopkeepers as a body arrive at the conclusion that the sale of spurious goods is against their own interests as traders. Nevertheless, it is very apparent that the practice of adulteration is far too prevalent. For instance, the report shows that in Scotland last year about 10 per cent. of all articles taken with the formalities required by the Sale of Food and Drugs Acts were found to be adulterated, while of samples procured informally no less than 17 per cent. were below standard. As regards England and Wales, the report shows that the number of samples taken was nearly a hundred thousand, but full particulars regarding the analyses of these are not yet available. It may be remembered, however, that in the previous year 95,664 samples of food and drugs purchased in England and Wales were examined by the public analysts and 8,169 were reported against. Since legal proceedings were instituted in only 3,643 cases and penalties imposed in 2,673, it may be assumed that in something like two-thirds of the total samples adversely reported upon, the extent of adulteration was not serious. The article which appears to be most frequently below standard is milk, but in this connection it is significant to notice the complaint of a dairy farmer in Essex, who called the attention of the Board to the fact that he was being prosecuted from time to time because his milk fell below the limit of 3 per cent. of milk fat referred to in the Sale of Milk Regulations, 1901, though the milk was stated to be sold in exactly the same condition as it was given by the cows. On inquiry by one of the Board's inspectors, it was ascertained that the milk in question was the mixed milk of a herd of some 50 cows of the cross-bred Dutch variety, which are reputed to be heavy milkers but to yield milk of somewhat poor quality at certain seasons. In connection with this case an officer of the local authority inquired if dairymen who kept only Dutch cows were to be allowed to supply milk that was considerably below standard, and in reply the Board pointed out that there is no legal standard of quality for milk, and that the Sale of Milk Regulations provide that, if milk is found to contain less than the percentages specified, it shall be presumed to be not genuine until the contrary is proved. If, therefore, the milk of Dutch cows is sold as given by the cows, the presumption that the milk is not genuine would be rebutted. A curious form of adulteration was brought to the attention of the Board by the circumstance that many creameries were approached by the makers of a highly refined form of cocoanut oil, who suggested that this oil might be used in connection with the manufacture of condensed milk. There is no ground for assuming that gross and fraudulent adulteration is in any way prevalent, but it is clear from the report that much remains to be done before a supply of genuine food can in all cases be assured.

